# A Website to Improve Asthma Care by Suggesting Patient Questions for Physicians: Qualitative Analysis of User Experiences

**DOI:** 10.2196/jmir.9.1.e3

**Published:** 2007-02-06

**Authors:** Christine W Hartmann, Christopher N Sciamanna, Danielle C Blanch, Sarah Mui, Heather Lawless, Michael Manocchia, Rochelle K Rosen, Anthony Pietropaoli

**Affiliations:** ^7^University of Rochester Medical CenterRochesterNYUSA; ^6^The Miriam HospitalProvidenceRIUSA; ^5^Health Dialog Analytic SolutionsBostonMAUSA; ^4^Wheaton CollegeNortonMAUSA; ^3^University of Massachusetts Medical SchoolWorcesterMAUSA; ^2^Brigham and Women’s HospitalBostonMAUSA; ^1^Jefferson Medical CollegePhiladelphiaPAUSA

**Keywords:** Asthma, Internet, qualitative research, patient education

## Abstract

**Background:**

Asthma is one of the most prevalent chronic conditions in the United Sates, yet despite the existence of national guidelines, nearly three fourths of patients with asthma do not have adequate control and clinical adherence to guidelines is low. While there are many reasons for this, physician inertia with respect to treatment change is partly to blame. Research suggests that patients who ask for specific tests and treatments are more likely to receive them.

**Objectives:**

This study investigated the impact and experience of using an interactive patient website designed to give patients individual feedback about their condition and to suggest tailored questions for patients to ask their physician. The website was designed to be used prior to a physician visit, to increase the likelihood that patients would receive recommended tests and treatments.

**Methods:**

A total of 37 adult patients with asthma participated in semi-structured telephone interviews aimed at eliciting information about their experiences with the website. Transcripts were coded using qualitative data analysis techniques and software. Themes were developed from subsets of codes generated through the analysis. In addition, 26 physicians were surveyed regarding their impressions of the website.

**Results:**

Opportunities exist for improving website feedback, although the majority of both patient and physician respondents held favorable opinions about the site. Two major themes emerged regarding patients’ experiences with the website. First, many patients who used the website had a positive shift in their attitudes regarding interactions with their physicians. Second, use of the website prompted patients to become more actively involved in their asthma care. No patient reported any negative experiences as a result of using the website. Physicians rated the website positively.

**Conclusions:**

Patients perceived that the interactive website intervention improved communication and interaction with their physicians, suggesting that patients can play a role in overcoming the clinical inertia of providers. Although the design and content of the website can be improved upon, the main findings suggest that use of the website is well accepted and is perceived to improve the quality of care that patients receive.

## Introduction

Asthma is one of the most common chronic conditions in the United States, yet it is estimated that approximately three fourths of patients with asthma do not have adequate control [1]. New interventions are needed to improve the care of patients with this condition [2-4]. In 1997 and 2002, the National Heart, Lung, and Blood Institute released guidelines for asthma care [5]. Despite the existence of these guidelines, studies show that health practitioners are not following the recommendations and that there is low compliance and inconsistency in asthma management nationwide [2,6,7].Noncompliance with guidelines can lead to overconsumption of health care resources, increased cost, and increased morbidity [8]. Though patient adherence to medications (eg, corticosteroid inhalers) is partly to blame, lack of asthma control also reflects “clinical inertia,” or the tendency of providers to make no treatment changes even though a patient has not achieved a treatment target [9-12]. However, research evidence strongly suggests that patients who ask their health care providers for tests and treatments are more likely to receive them [13,14], though the effect of this strategy on chronic disease management has not been well studied [15].

**Figure 1:**
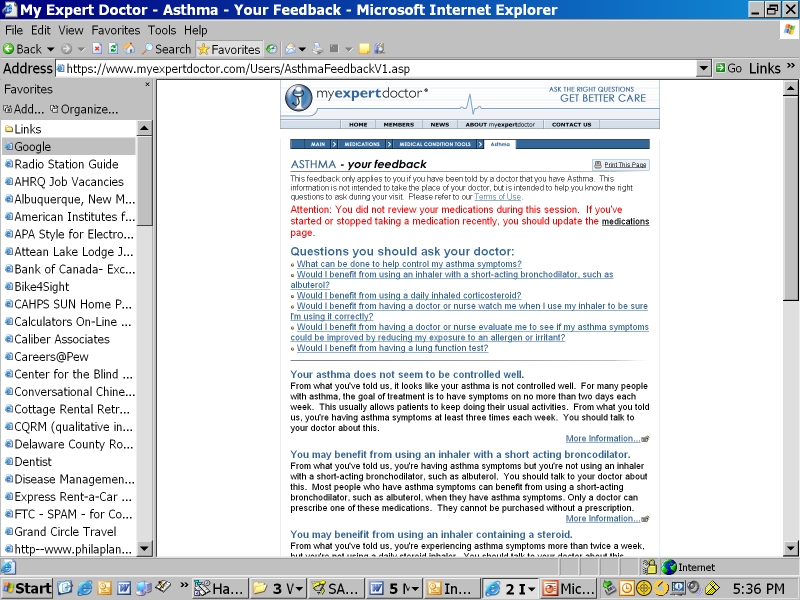
Sample questions for physician and tailored feedback

To test the impact of patients asking their health care providers about tests and treatments they could receive, we developed an interactive website (myexpertdoctor.com) to inform patients about asthma and to provide tailored feedback. The website is designed to be used before a physician visit to help patients know what questions to ask during the visit, which in turn may increase the chance that they receive tests and treatments suggested by evidence-based guidelines (see Figure 1 and the Multimedia Appendix for sample screenshots).

Computer applications have been used to improve asthma control by improving patient education [16,17], disease monitoring [18,19], and by prompting physicians to practise guideline-concordant care [20-22]. However, we are not aware of any interventions designed to prompt patients to ask questions during provider visits in order to improve the quality of their care. We conducted a qualitative study to understand the effects of a Web-based intervention on the physician-patient relationship and on asthma care [23,24]. Although previous studies have shown that the intervention did, by pointing out deficiencies in the quality of their care, cause users to believe they received worse care [5,26], data are needed to more fully understand the potential effects of such an intervention, in particular, how the intervention can impact doctor-patient communication. That was the goal of the current study.

## Methods

### Intervention Development

The overall design of the Web-based intervention, which included modules related to various medical conditions, including migraine and osteoarthritis, has been published elsewhere [5,26]. To review, four steps were used in developing the intervention. First, evidence-based decision rules were identified by reviewing clinical guidelines [5]. Second, a self-report survey was created to measure the adherence to each of the guidelines. Next, tailored feedback and suggested questions were created and prioritized for each guideline (see Table 1 and the Multimedia Appendix). Finally, the questions and feedback were programmed into a secure and reliable website, resulting in a three-step process for study patients accessing the site prior to a physician visit. First, patients were prompted to answer 10-20 questions relating to their asthma and its care. Next, patients received immediate personalized feedback and information about their condition, including a list of suggested questions to ask their physician. And finally, patients were encouraged to use this information and the questions during their upcoming physician visit.

**Table 1:** Website feedback and suggested question examples

**Feedback for Care Not in Keeping With the Guideline**	**Suggested Question to Ask Physician**
**You may benefit from using a corticosteroid pill for your asthma.**From what you told us, your asthma is “worse than usual.” You also told us that you’re not taking a corticosteroid pill. You may want to ask your doctor about this. Some doctors choose to add a steroid inhaler instead. Corticosteroid pills are very helpful when asthma symptoms are worse than usual. They can help you to feel better much more quickly. These steroids are not bodybuilding steroids. In general, they should only be taken for a few days. (Click here for more information.)	Since my asthma is worse than usual, would I benefit from taking a steroid medication by mouth?
**You may benefit from using an inhaler when you feel short of breath.**From what you told us, you have some symptoms from your asthma. You also told us that you don’t have an inhaler to use when you feel short of breath. You may want to ask your doctor about this. There are several good inhalers for when you’re short of breath, such as albuterol. These inhalers work great to make you feel better quickly. (Click here for more information.)	Would I benefit from using an inhaler with a short-acting bronchodilator, such as albuterol?
**You may benefit from using a steroid inhaler.**From what you told us, your asthma is not well controlled and you’re not using a steroid inhaler. You may want to ask your doctor about this. Steroid inhalers help to prevent you from feeling short of breath. Steroid inhalers are used every day to prevent asthma symptoms. A steroid inhaler is a very effective medicine for asthma. They’re not like bodybuilding steroids and are quite safe.	Would I benefit from using a daily inhaled corticosteroid?
**You may benefit from using a second inhaler to prevent asthma symptoms.**From what you told us, your asthma is not well controlled. Also, you’re not using a second type of medicine to prevent asthma symptoms. You may want to ask your doctor about this. Medicines that prevent asthma symptoms come in two types. The first are steroids, such as Beclovent. The second type opens the airways, such as Serevent. Both of these medicines should be used each day, whether you have symptoms or not.	Would I benefit from using a long-acting bronchodilator like salmeterol?
**You may benefit from using a peak flow meter at home.**It’s not always easy to know when your asthma is getting out of control. From what you’ve told us, you’ve been to the emergency room at least a couple of times over the past year. Because of that, you might benefit from using a peak flow meter every day. You may want to ask your doctor about this. A peak flow meter is a small plastic tube that you blow in to see how your asthma is doing. That way, if you feel okay, but your peak flow is low, you can make a change before you feel worse. It’s also important for you to know what to do depending on your peak flow number. This is also something to discuss with your doctor. This is where an “asthma action plan” comes in handy. This is discussed below.	Would I benefit from using a peak flow meter to monitor my asthma at home?
**You may benefit from seeing an asthma specialist.**From what you’ve told us, you’ve been to the emergency room at least a couple of times over the past year. Because of that, it’s important for you to see a specialist once in a while. You haven’t seen an asthma specialist in the past year. You may want to ask your doctor about this. A specialist can help you figure out if you need different tests or treatments for your asthma. These doctors include “pulmonologists” and “allergists.”	Would I benefit from seeing an asthma specialist at this time?
**You could do better to prevent asthma attacks.**From what you told us, you’re using a medicine to prevent asthma attacks. These are called “controller” medications, such as the Azmacort that you are taking. You are not using your controller medicine every day. You may want to talk to your doctor about this. Controller medications help to prevent you from feeling short of breath. They need to be used every day, even if you feel fine.	How frequently should I be using my controller medication(s)?

The website feedback consisted of three elements: (1) a list of suggested questions for the patient to ask his or her physician, (2) a lay explanation of why the patient should ask the physician these questions (one message for those whose care was in keeping with the guideline [eg, moderate persistent asthma whose medication list included a corticosteroid inhaler] and one for those whose care was not), and (3) links to other websites for further reading and explanations of the suggested topics (sites selected and reviewed by panel of experts). By pointing out areas for potential improvements in care (“quality gaps”), we had a concern that some patients would believe that their provider was not giving them needed care. We expended efforts to make the feedback as neutral as possible with regard to this issue. For example, rather than indicating “you need…,” the feedback typically suggested “you may benefit from….” The rationale for the feedback was based on the Chronic Care Model, a theoretical model of chronic illness care in which one of the overarching goals is to foster productive interactions between patients, who actively participate in their care, and providers, who can draw on the expertise of guideline-based reminders [27,28].

### Study Design

The study was designed to document the experiences of patients who had used the asthma quality improvement website prior to a visit with their asthma care provider. Because qualitative methods are useful when conducting exploratory research [23,24], and little is known regarding the impact of these types of interventions on quality of care or on doctor-patient communication, this study employed a semi-structured interview methodology to investigate the range of patient experiences with the website, before and during their doctor visit [29]. The interview guide was created by the research team, including two doctoral-level anthropologists and a board-certified internist (CS). Interview questions focused on patients’ impressions of the utility of the website, including their experiences using it and specific website navigation issues, as well as the effect of the tailored feedback from the website on doctor-patient communication and perceived quality of care during patient visits. In addition, physicians were recruited to view the website and subsequently participate in a survey regarding their impressions of the site. Ethical approval for the study was obtained from the Institutional Review Board at The Abacus Group, LLC, Cranston, RI, USA.

### Sampling and Recruitment

In response to an advertisement on Google and letters mailed to asthma patients of a large health insurance company in Rhode Island, USA, potential subjects were encouraged to contact research staff and were screened over the phone for inclusion and exclusion criteria. The following inclusion criteria were used: (1) age greater than 21 years, (2) self-reported history of asthma, (3) a planned visit with an asthma care provider in the next 2 months, (4) Internet access at home or work, and (5) asthma care from a primary care provider rather than a specialist (eg, pulmonologist). During the screening phone call we asked patients the date of their next asthma provider visit and set a date for a follow-up phone interview after the visit. The final sample comprised 37 patients, who were mailed an informed consent form. The content of the form was explained over the phone, and subjects were encouraged to call the research team if they had any questions. All 37 subjects returned the consent form by mail before the date of their physician visit. Subjects were reimbursed US $100 for participation in the study.

For the physician sample, a nationally representative database of primary care providers was purchased from a marketing organization. A recruitment letter was mailed to a national random sample of 250 physicians; 26 physicians agreed to participate in the study and completed the anonymous survey on the study website. Physicians were reimbursed US $100 for participation in the study.

### Data Collection

 A research assistant monitored patients’ use of the website to be certain they used it before their physician visit. Emails were sent to every participant 7, 4, and 3 days before the date of the visit, reminding them to use the website. Two of the 37 subjects had not used the website within 72 hours of their planned asthma care provider visit and were called by the research assistant to remind them to use the website before their visit. Prior to the asthma care visit, all 37 subjects used the website, answering questions and receiving personalized feedback. The website and the research assistants encouraged patients to print the individualized feedback and take it with them to the physician visit. After visiting their physician, all patients were contacted and participated in a semi-structured telephone interview that lasted approximately 25 minutes. The interviews were conducted by one of two trained research assistants (DB, HL) using an interview guide created by the research team. Open-ended questions, through the use of follow-up questions and probes, allowed for in-depth exploration of topics such as those related to the individualized feedback provided by the website, how this feedback shaped (if at all) the participants’ interactions with their physicians, and whether and how the website and feedback were useful in helping them communicate with their physician [29]. Close-ended questions were also asked and addressed (1) use of the website before the visit, (2) use of the website information during the physician visit, and (3) how use of the website and feedback changed the physician visit, if at all.

Physician data were collected via a brief online survey posted on the study website. Physicians were asked to provide written answers to three close-ended and three open-ended questions regarding their impressions of the website, their perceptions of its usefulness to patients, and their suggestions for improvement.

### Data Analysis

Patient interviews were audiorecorded, transcribed, and entered into QSR NVivo qualitative software (version 2.0; QSR International; Melbourne, Australia) in order to facilitate data management and analysis. The transcripts were coded by a doctoral-level anthropologist based on the grounded theory technique, in which codes are drawn from the text and coding involves frequent comparative analysis of the data [30,31]. In order to establish the coding scheme, a random 25% of the interviews were coded by an additional doctoral-level anthropologist and discrepancies in coding were resolved via consensus [24,31,32]. Overall, 108 separate codes were identified. As this was a pilot study, a majority of the interview questions, and therefore the codes, related to patients’ experiences using the Internet for medical searches and their impressions and use of specific pages of the website. For purposes of the thematic analysis presented here, we were interested in understanding the experiences of patients using the website and how use of the website impacted their physician visit, with particular attention on the doctor-patient interaction and relationship. For that reason, in the thematic portion of the manuscript, we concentrate on presenting the codes and themes that were germane to this issue. Patients’ suggestions for improving the website are presented as well. Physician data were collected online and transferred to an Excel database. All descriptive statistics were calculated using SAS (version 9.1).

## Results

### Quantitative

The large majority of the patients in this study were female (34/37, 91.9%). Approximately one fifth were under the age of 35 (8/37, 21.6%), and only four patients were over the age of 60. The majority were white (33/37, 89.2%), and 45.9% (17/37) had completed college. Asthma symptoms were experienced three or more times a week by slightly more than four fifths of the patients (30/37, 81.1%). In keeping with data that show younger people are more active in terms of using the Internet to seek out health information [33], 67.6% (25/37) of the patients in this study indicated using the Internet at least once a week for this purpose.

All patients (N = 37) used the website before the visit with their physician; however, only 36 patients answered the questions regarding use of the website. While most of the patients (26/36, 72%) indicated that they brought a printout of the website feedback to the visit, overall only slightly more than half (21/36, 58%) told their physician that they had visited the website. When asked whether use of the website had influenced the outcomes of their visit in any way, 56% (20/36) answered affirmatively. However, with regard to this response, it is important to note that when patient experiences were explored in-depth, almost all patients indicated that use of the website had influenced the visit in some manner. Most participants rated the website positively: excellent, 25% (9/36); very good, 55.6% (20/36); good, 16.7% (6/36); fair, 2.8% (1/36); and poor, 0%.

With regard to the physician (N = 26) responses to the two close-ended questions pertaining to impressions of the website, a large majority of the physicians rated the website positively: excellent, 11.5% (3/26); very good, 50% (13/26); good, 23.1% (6/26); fair, 11.5% (3/26); and poor, 3.8% (1/26). In addition, slightly more than three quarters (20/26, 76.9%) responded positively to a question asking whether they thought that the website and its feedback would be useful in helping patients receive better health care. No demographic information was collected from physicians.

### Qualitative

Overall, patients in this study found that having information from the website positively impacted their interactions with their physicians, and, importantly, while some patients reported some dissatisfaction with the website overall, no patient reported a negative encounter with the physician attributable to use of the website. Physicians also reported positive feelings about the website content, while at the same time offering suggestions for improvement.

### Physician Impressions of the Website

Physicians were asked three open-ended questions regarding (1) what they thought of the website’s feedback section, (2) what suggestions they had for improving the website, and (3) what other comments they had about the website. There were 22 respondents. Physicians’ impressions were very favorable regarding the first question and are not summarized here. However, physicians’ responses to the other two items did highlight a number of opportunities for improvement.

The most common comment (n = 10) had to do with providing more specific information about medications:

Physician 16 Provide info and precautions concerning specific medications.

Physician 25 You may want to avoid using the term “steroid.” Perhaps tell the patient that a medication such as prednisone or Medrol might help their exacerbation. In my experience the term "steroid" can be a big turnoff despite trying to educate the patient.

Some physicians (n = 5) believed that the website could be strengthened through inclusion of even more specific information about asthma:

Physician 41 [Include] more patient education regarding control of triggering factors.

Physician 9 [Include] some basic information about the physiology of asthma that could explain how medications work, so that patients are more motivated to take their medications when needed.

Others (n = 3) suggested the addition of more visual aids:

Physician 24 A printout or prototype of an asthma management plan for the patient to review prior to physician consultation may be helpful. Also, charts of predicted peak flow values would likely be more educational for patients than written explanations of normal values.

Physician 55 [Include a] visual/schematic of peak flow, spirometry.

A number of other comments fell into no specific category and ranged from encouragement to keep the questions and feedback simple, to providing lists of asthma specialists in the area, to allowing for questions from patients and including an appointment schedule.

### Patient Suggestions for Improvement of Website

A total of 37 patients suggested improvements for the website. Their feedback pertained to its not providing enough feedback, not providing new information, not giving feedback that was specific to the user, and not having enough scientific information. For example, some participants (n = 13) found that they wanted more detail in both the questions and the feedback, particularly some of the participants whose asthma was rated by the website as being “under control.”

Patient 336: female, age 34 [The website] didn’t come up with any questions or anything to ask my doctor. It pretty much just said, “Oh yeah, your asthma is under control.”

Patient 309: female, age 36 I expected more detailed questions [in the feedback section]. It seemed pretty general.

Another type of comment had to do with the website’s feedback not providing enough information that was new (n = 10). These participants were knowledgeable about their condition because they had either had the condition for many years or had spent time researching it using either the Internet or other means.

Patient 338: female, age 33 I’ve done so much research already that most of what it was telling me, I already knew.

Patient 321: female, age 35 I go to other websites and read about it to learn about it and um...I think if a website is gonna be used to help people ask questions, I think there should be a little bit more information about the asthma itself on the site.

In addition, some users (n = 5) perceived the feedback as not being specific enough to their own situation. While this opinion was only held by a minority of participants, these users felt that the feedback either too closely mimicked the original questions that had been asked or that the feedback was too general to fit their specific situation.

Patient 359: female, age missing It may be that 90% of the people that have asthma fall into the categories that this would help, but I think I might be in the 10% that would have...a different kind of experience of asthma than the other 90%.

Patient 326: female, age 41 When it gave me the questions and gave me answers that they think I needed to look into, it basically said the same thing again.

A final small group of users (n = 2) perceived the feedback as being not scientific enough, by which they meant not having enough information about current asthma research.

Patient 355: male, age 30 I think it would have been good if it [the feedback] had said here is what asthma research is going.

Patient 305: female, age 26 For me more scientific information may have been useful...like if it [the feedback] gave links to publications for epidemiological studies or drug studies.

A majority of the users who expressed some reservations about the website nevertheless found some aspect of it to be helpful, either the feedback, the links to other sites, or simply the encouragement to approach their physician with questions.

### Patient Themes

Patients’ answers to interview questions regarding the individualized feedback and the impact of the website itself centered around two main themes. First, one common result of visiting the website and subsequently visiting a physician was a positive shift in patient attitudes regarding interactions with their physicians. Patients reported that they had more self-confidence, they talked more during the visit, and they had more confidence in the care they were receiving. A second, related theme focused on patients becoming more actively involved in their own asthma care. They gained a better understanding of their treatment options and of their role in managing the condition.

### Theme 1: Positive Shift in Patient Attitudes Toward Interactions With Physician

A majority of patients (20/37, 55.6%) answered “yes” when asked a close-ended question about whether the website had influenced their physician visit in some way. In addition, many of those answering “no” or “unsure” to the same question nevertheless revealed in answers to subsequent open-ended questions that their visit had been positively affected. Common to many patients’ statements was an increase in self-confidence with regard to communicating with the physician because of the information and questions from the website.

Patient 308: female, age 49 I’ve been going to this doctor for about 17 years, [but this was] the first time that I’ve actually gotten anywhere with him as far as changing what he was doing for me.... [The website gave me answers to] a lot of the questions that I had in the back of my mind as to why my doctor wasn’t pursuing a different avenue. That was kind of cleared up for me, and [it] also gave me the questions to ask him that seemed to push him in the right direction as far as giving me something on a daily basis instead of the inhaler that I was becoming reliant on.

Patient 344: female, age 49 [My asking questions and having information] threw my doctor for a loop ‘cause it took him by surprise. [Laughs.] But it was a good thing. It was a real positive thing. It actually allowed us to create a stronger working relationship with controlling my asthma.

Use of the website also increased the amount of time patients talked during the physician visit. Some patients took the feedback printout with them and used it during their physician visit, while others read it before the visit to remind themselves of the content. Because patients were familiar with the suggested questions on the printout and felt secure about what they wanted to ask their physician, they felt more relaxed and interjected more; this, in turn, had a positive impact on the impression of their interaction with the physician, independent of the answers they received to the questions.

Patient 313: female, age 35 [You normally] have the questions that you want to ask, but you don’t write them down or if you’re really sick then you’re not even thinking about it. You’re just trying to get there. Your stress level’s pretty high. [With the printout], I was relaxed a little bit that I was already ready, and I didn’t have a lot of work to do. I can just grab the paper and go. It made it a little bit.... [pause] There was a little more discussion with my doctor about my asthma and a couple of triggers. We talked more.

Patient 340: female, age 42 If I hadn’t had that piece of paper [the feedback form] in my hand, I think I would have just gone as a regular office visit and just sat there and waited for her to make a decision, without contributing. [This time] I was able to speak about the fact that I probably should—or she probably should—look at other means of treatment, and that’s different than my usual office visit where I don’t make any suggestive contribution to what part of treatment is. I just take it all in.... [This time] I was part of the discussion, part of my treatment, and part of the whole total picture of getting my prescriptions changed.

Finally, because the website was able to give them or guide them to specific information about their condition, patients repeatedly mentioned feeling more confident in their understanding of issues related to their asthma and its treatment. This led to what they perceived as improvements in the physician-patient relationship and greater confidence in the care they were receiving. In some cases, the physician’s authority was validated by the patient’s own research, rather than the research being validated by the physician’s authority. This was in part due to the fact that the website and the links it provided gave more information than these participants felt they could glean from their short interactions with their physicians.

Patient 315: female, age 26 [The feedback form] gave me an opportunity to start off with some questions.... So I had some kind of say in what was going on. Because when I first went in, he just suggested medications for me and everything else, and I had no clue what the medications were, what they did, side effects, or anything like that. So, at least this way it gave me a little advantage because I knew from reading over the information before I went in kind of what the treatment options were.

Patient 313: female, age 35 [The information] helped a little bit, that I might actually be building this stronger relationship with my doctor by initiating conversation and questions for him.... I was able to talk to him and feel more confident in the treatment that I’m receiving.

Patient 304: female, age 47 [The website] listed six or so different [asthma] symptoms, and one of them I don’t have and I guess most people do, but I could see that it doesn’t have to only be this. So it makes me more confident that what my doctor told me is accurate.... [It] validates what the doctor told me.

As can be seen from the quotations above, use of the website promoted affirmative changes in patient attitudes toward their interactions with their physician. Not only did visit experiences improve from the patients’ perspectives, but some patients also indicated that their questions prompted changes in their treatment. In other words, asking specific questions was reported to overcome clinical inertia and result in positive modifications in patients’ care.

### Theme 2: Patients Becoming More Actively Involved in Their Asthma Care

Participants varied in how they spoke of their knowledge about asthma and their understanding of their role in managing their asthma. A number of patients were generally more knowledgeable about their condition than others, and while some found the information on the website too limited in scope, others thought the website encouraged them to actively seek additional information, either online, in print sources, or from other individuals. Some patients had traditionally relied in part or, to a lesser extent, exclusively on their physicians for information about their treatment. For many of these patients, the information provided in the feedback and from the links to other sites led to an increased understanding of their condition and of how they could become more involved in their own care.

Patient 318: female, age 41 [The website feedback included] questions I never thought to ask. One of mine was about asking “Should I be taking steroid medication by mouth?”.... I had to do some research on what they considered steroid medications.... [That helped me] to think more about how to take steroids and myself and more questions to ask the doctor.

Patient 309: female, age 36 [After looking up information on an in-home peak flow meter:] I didn’t know that people had those at home at all. I didn’t know that that would be something I could do that would help monitor [my asthma]. That was the major thing. I didn’t really have a good idea of how sick I was.

For both well-educated and less-informed patients, having access to specific information about their condition frequently resulted in the decision to become more actively engaged in their care. What they learned through having used the website led to changes in how they wanted to approach their care. Patients became more aware of treatment opportunities and the need for changes in the frequency or style of their involvement in managing their condition.

Patient 326: female, age 41 I liked that it [the website] told me that maybe I should be taking more precautions and looking more into my [asthma], and maybe I’m not controlling it as well as I should, that maybe more attention should be brought to it each day instead of like once a week, twice a week.

Patient 344: female, age 49 [Asking questions from the feedback sheet] creates a relationship where you’re working together to create a plan, and it’s not just the doctor creating the plan.... I have more knowledge now to be able to go to him and have him work on me.

Patients’ responses illustrate increased involvement in their own care as a result of information and feedback attained either directly or indirectly through use of the website. In addition, as was evidenced in the first theme, having patients pursue new treatment alternatives and become more actively involved in their own care led to perceived positive changes in physician behaviors, including medication and monitoring adjustments.

## Discussion

Inconsistencies in the implementation of and noncompliance with asthma treatment guidelines contribute to a reduction in health quality for individuals with the condition [1,34]. This study of a novel Web-based intervention investigated the impact of giving patients individualized information that was designed to prompt physician-patient discussions around issues raised by the evidence-based asthma guidelines [35] and improve the quality of care. To date, to our knowledge, no research has been performed on this type of strategy. Analysis of the data collected showed that knowledge gained from the website and its feedback form positively influenced patients’ interactions with their physicians, their knowledge about asthma, and their feelings of responsibility for managing their condition. In addition, patients accepted and enjoyed using the technology to assist with their asthma care, and physicians had positive impressions of the site and its potential to improve care.

Our qualitative results validate the findings of many quantitative studies that have examined similar issues using survey data. Our previous research has shown that patients have a need for the information that is provided on the website. For example, in 2001, we asked 300 primary care patients if they had ever used or had any interest in using the Internet to “find out what questions you should ask your doctors when you see them” [36]. Nearly 60% of the patients were interested in using the Internet for this purpose, and fewer than 30% of the patients had ever found this information on the Internet. It is therefore not surprising that such a high percentage of patients in this study were satisfied with the asthma website. In a similar survey-based study, in which patients were observed searching the Internet for health information before a doctor visit, most (90%) reported feeling more satisfied with their visit than with previous visits because of the Internet use [37]. In that study, patients were trained to use a specific website with links to specifically chosen patient education websites. Those positive findings, coupled with our current findings, suggest that a guided Internet search experience can be quite acceptable to patients and can improve satisfaction with subsequent doctor visits [37].

We hoped that by providing patients with a small number of evidence-based questions, they would have an enhanced patient-provider experience. A previous review of the effects of the Internet on doctor-patient communication identified some concern that physicians may be annoyed by patients bringing in information from the Internet, and that this may possibly harm the doctor-patient relationship [38]. For example, only 15% of 168 physicians surveyed believed that the Internet would improve their relationship with patients, while 49% felt it may harm it [39]. Our results generally point toward the intervention helping patients to communicate better with their physician in order to become more of a partner in their own care. No patient mentioned having a negative interaction with the physician as a result of having used the website. Additionally, the physicians themselves rated the website positively and indicated that they thought the website would be useful in helping to improve patient health care.

We were pleased that subjects viewed the website as reinforcing the care they were receiving from their physician, rather than causing them to question it and potentially undermining their belief in their physician. Our findings are consistent with two other published studies of the website, which observed that the website feedback had no adverse impact on patient perceptions of overall quality of care from a physician [25] or care during a physician visit [27]. This is also consistent with the findings of Kivits [40], who observed that patients viewed Internet health information as complementing, rather than opposing, information from their doctor.

### Limitations

Data in this exploratory study were collected from a convenience sample of individuals—the majority of the patient participants were white, female, and over age 35. Because of the sampling method, the results may not be generalizable to patients with asthma, the general patient population, or the physician population. In addition, patients were prompted several times to use the website before their provider visit, including phone calls, which would not occur if the website were implemented in a nonstudy setting. However, although research in the area of using websites to impact physician-patient interactions is new, previous research does support patients’ positive reactions to prompts designed to further physician-patient communication [41,42].

Because some of the patients’ physicians may have seen the printout from the website and some patients may have told their providers that they were participating in a study, a Hawthorne effect cannot be ruled out. Nevertheless, as the intention of the study was to have patients use the information from the website to prompt discussions with their physicians through the use of the feedback form, we judged the bias attributable to this effect to be limited. Although some providers may have known that their patients were study participants and therefore may have paid more attention to the patients’ questions, none of the data collected from the patients themselves indicated that this was the case.

Finally, although patients reported changes in the interactions with their physicians, these may not have translated into changes in patient care. Further research needs to be conducted on whether the website is successful in improving patient outcomes.

### Implications for Practice

The Internet has great capacity to positively influence health care, and there is good evidence that it is already an entrenched part of the medical landscape. In the United States, 60% of adults have Internet access, and over 80% of patients with Internet access have searched for health information online [33]. Similarly, we have observed in a survey of 330 primary care patients that most (62.1%) patients felt that their doctor should “recommend specific websites where I can learn more about my health and health care” [43]. However, once physicians encourage patients to use the Internet, patients are likely to hold them accountable for discussions of the information they find. In a separate study, we observed that patients whose physicians did not discuss information gleaned from a tailored-message computer program, much like the website we have designed, were significantly less satisfied with their visit [44]. This suggests that there are likely to be bumps along the road in making suggestions from websites and discussions of Internet searches a part of routine care.

Nevertheless, having patients ask the most pertinent questions relating to their care may help them get the most out of the normally brief office visit. Having patients access this type of information while in a doctor’s office has been shown to be too challenging [45]. And while lists of questions to ask a physician about specific conditions are available online (from the American Heart Association, for example), the website in this study took these types of questions one step further, tailoring the feedback based on individuals’ answers and explaining why each feedback question was important. In addition, the feedback page placed questions about controlling the condition at the top because results from a prior study indicated that patients were more likely to ask the questions at the top of the feedback page [26]. It is possible that interventions such as the one in this study may increase the efficiency of brief office visits by allowing patients to access pertinent information at home and come to the visit prepared with a list of individualized, prioritized questions and a greater understanding of why asking these questions is important.

While the website has clear implications for practice, it was not designed to be made available to patients in a physician’s office. Rather, it should be viewed instead as a prototype for possible distribution through a number of channels: (1) managed care organizations, to improve the quality of care they provide and satisfy accreditation requirements from the National Committee on Quality Assurance [46]; (2) employers, to decrease work limitations from chronic diseases such as asthma; and (3) advocacy groups, such as the American Lung Association, to improve the quality of care for their constituents. Being a narrowly focused website, it is a relatively inexpensive intervention that requires minimal maintenance, as guidelines are not published that often and major changes to standards of care occur infrequently. Given the steady increase in Internet access [33], we believe that future versions of myexpertdoctor.com and similar offerings could have a significant and positive impact on asthma care and quality of life among patients with asthma and other chronic conditions. 

### Conclusions

The present study has given us confidence that the current intervention has the potential to improve the way patients communicate with their provider and that the suggested questions can overcome the clinical inertia of providers. Both physician and patient users of the site provided useful feedback on changes that could be implemented in future versions of the website to make it more effective. The main findings—that use of the website and its feedback form positively influenced patients’ interactions with their physicians, their knowledge about asthma, and their feelings of responsibility for managing their condition—all point in the expected direction and suggest that the website can improve the quality of care patients receive. We believe that it is essential to give the Internet functionality beyond being a passive, albeit massive, repository of health information. A national study of 4764 adults who used the Internet for health information noted that only one in six believed that the Internet had influenced treatments that they used for a health condition [47]. Although patients may have the potential to learn a great deal, much of the information is beyond their comprehension as it is written at a high reading level and many patients are relatively health illiterate [48,49].

However, empowering patients with specific questions to ask appears to put health information into patients’ hands in a way that activates them to be involved in their care. Despite the great number of medically oriented websites, we are not aware of another that provides patients with evidence-based questions to ask their doctor. Most interactive health websites focus instead on providing tailored risk assessment, such as the Heart to Heart Tool [50], Heart Profilers on the American Heart Association website, RealAge, and WebMD. Ongoing studies are evaluating the effect of the website we have designed on patient health outcomes. Given the steady increase in Internet access, we believe that if the current intervention proves to be effective, it may have a significant impact on the control of asthma, as well as other chronic medical conditions.

## Multimedia Appendix

Screenshots of the website
