# Supplementary figures and images for: A Website to Improve Asthma Care by Suggesting Patient Questions for Physicians: Qualitative Analysis of User Experiences
Source: J Med Internet Res. 2007 Feb 6;9(1):e3. doi: 10.2196/jmir.9.1.e3 (PMC1794671; doi:10.2196/jmir.9.1.e3)

## Slide 1
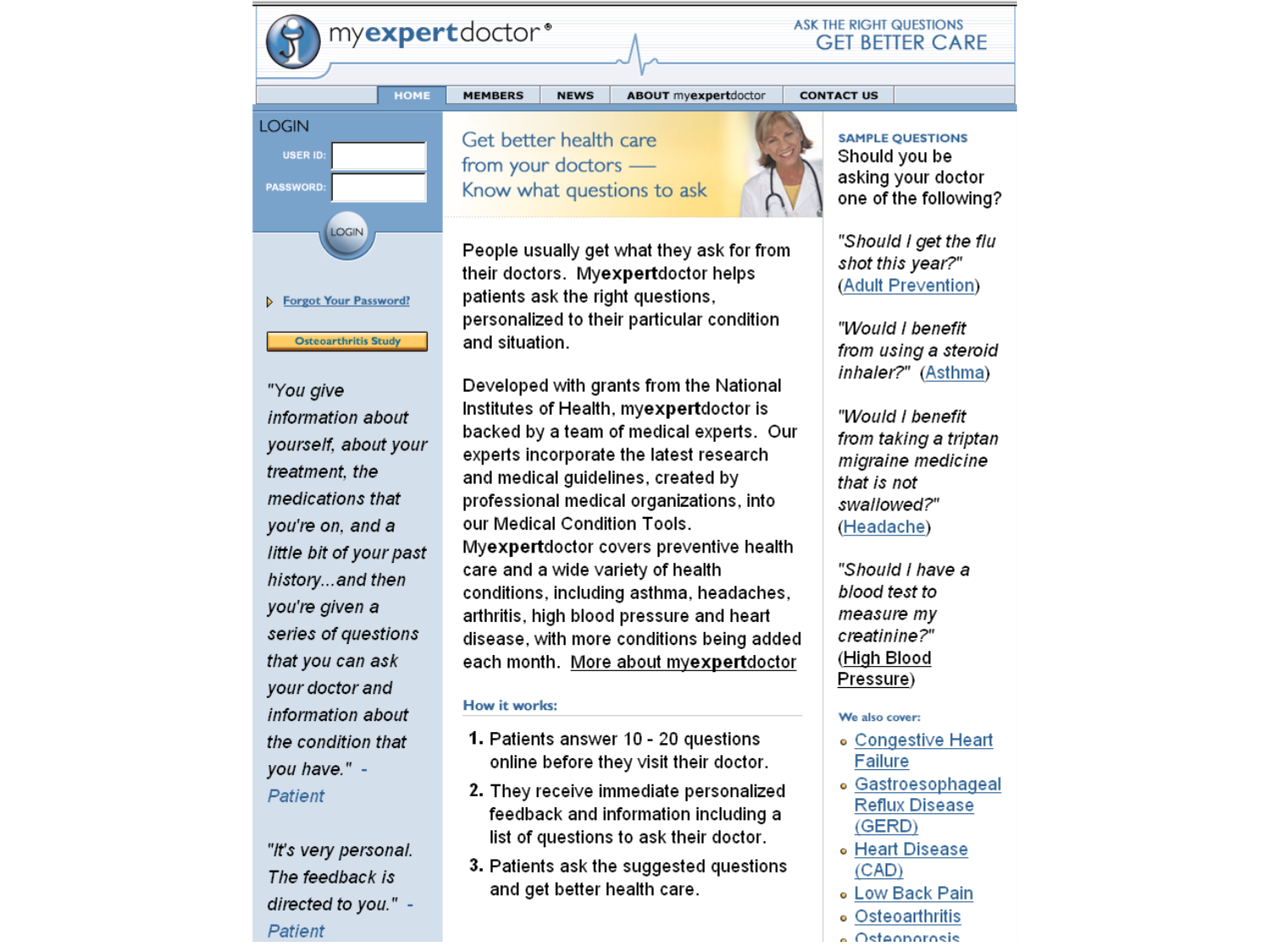

## Slide 2
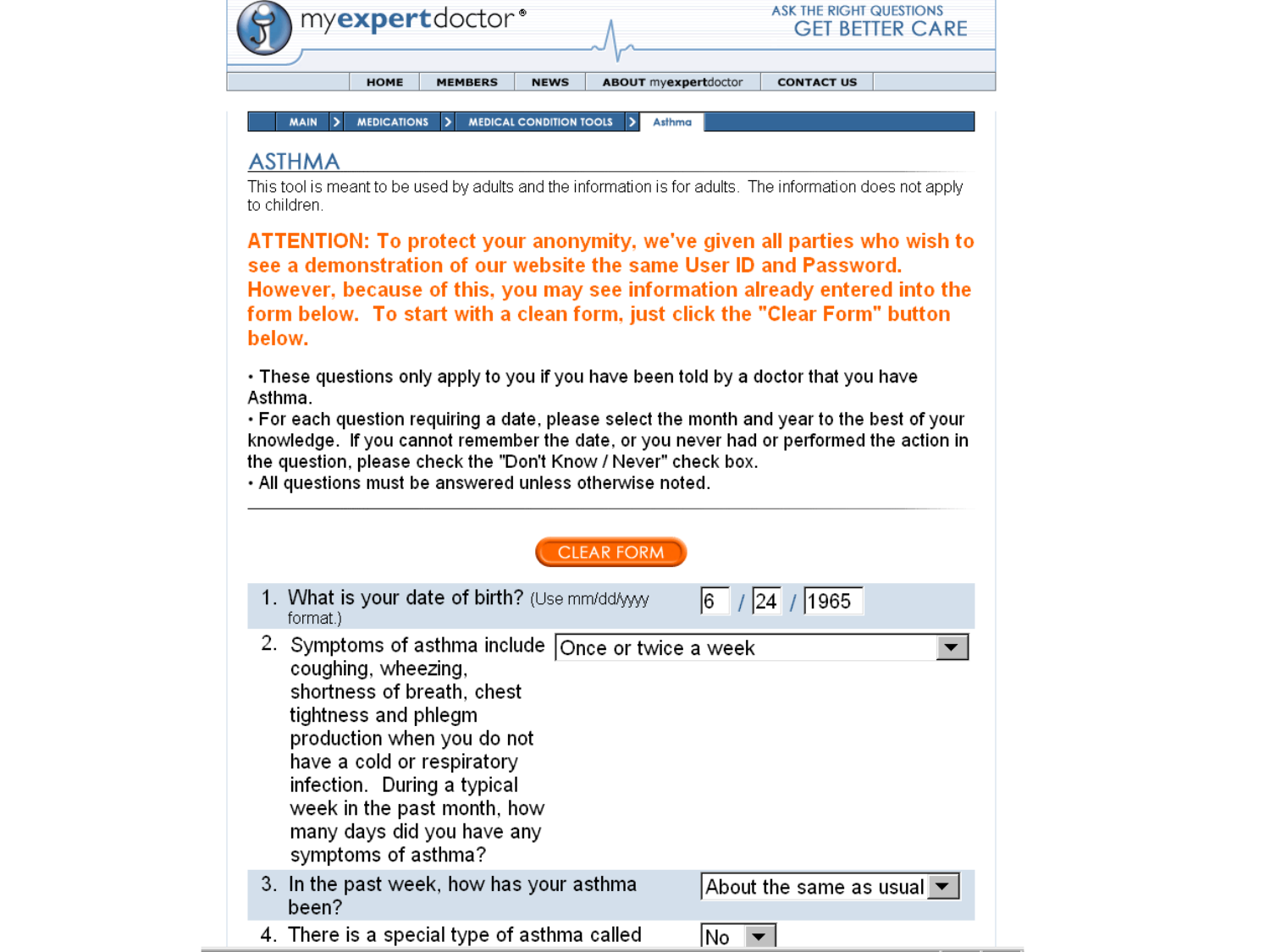

## Slide 3
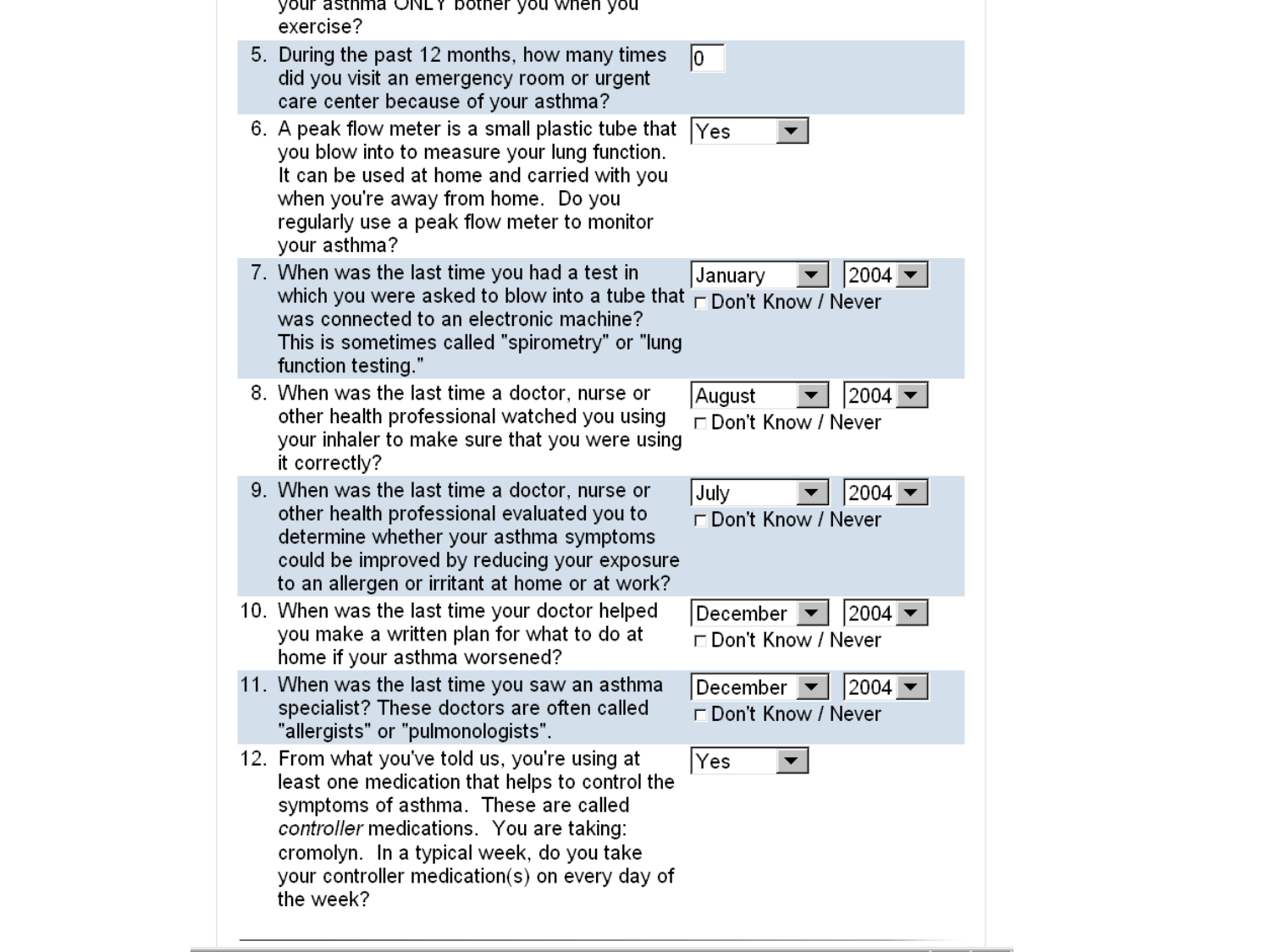

## Slide 4
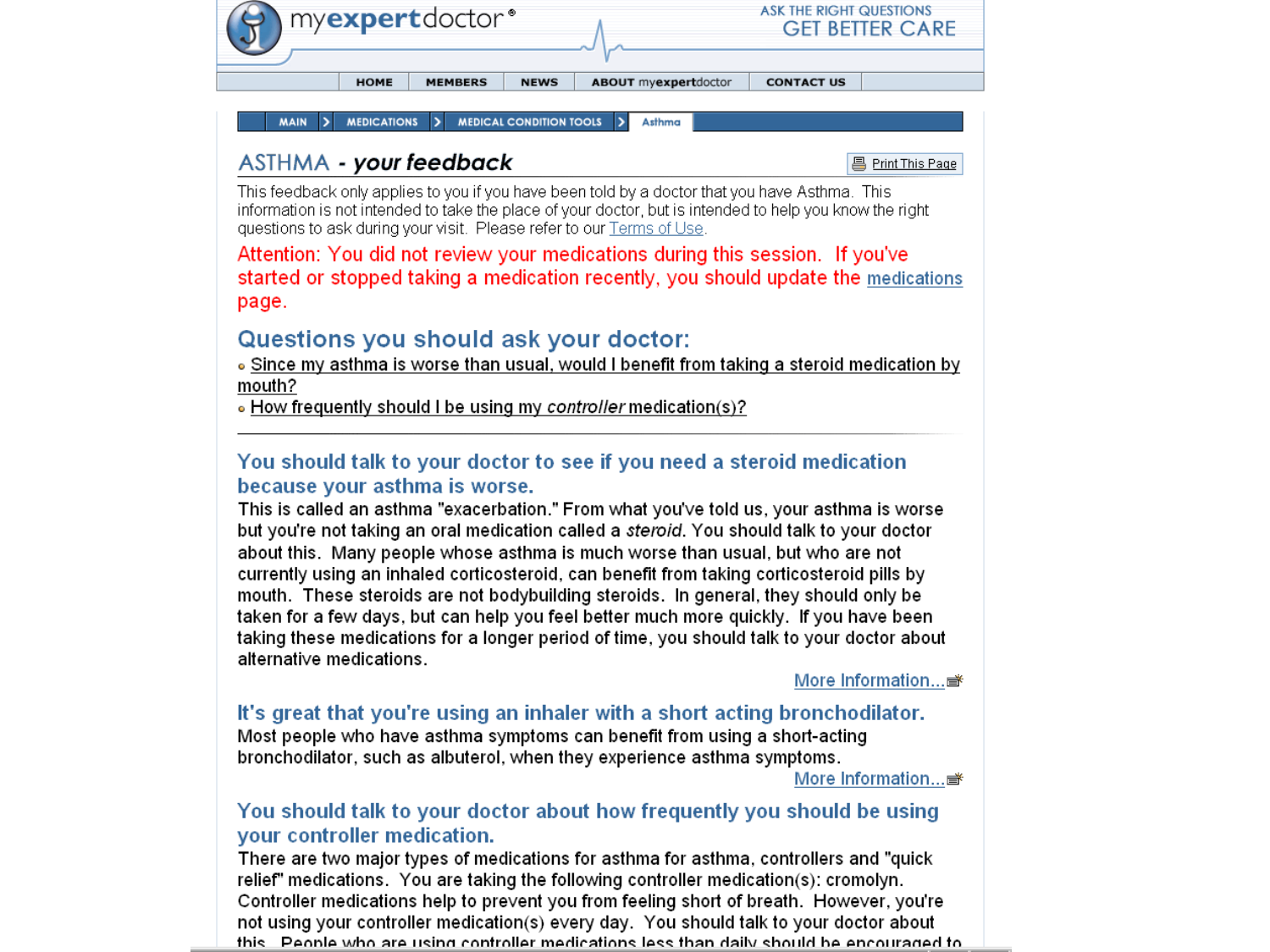

## Slide 5
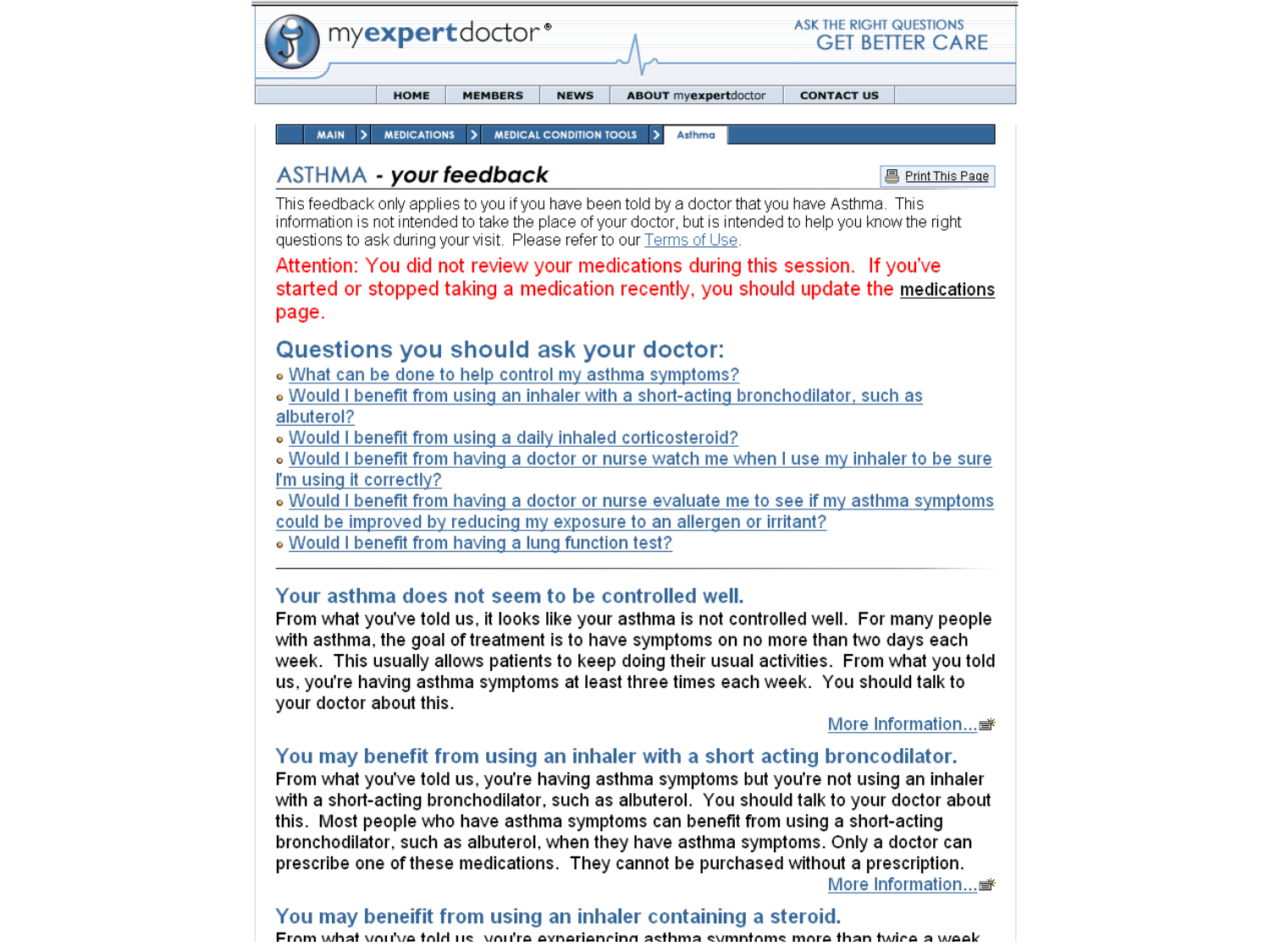

## Slide 6
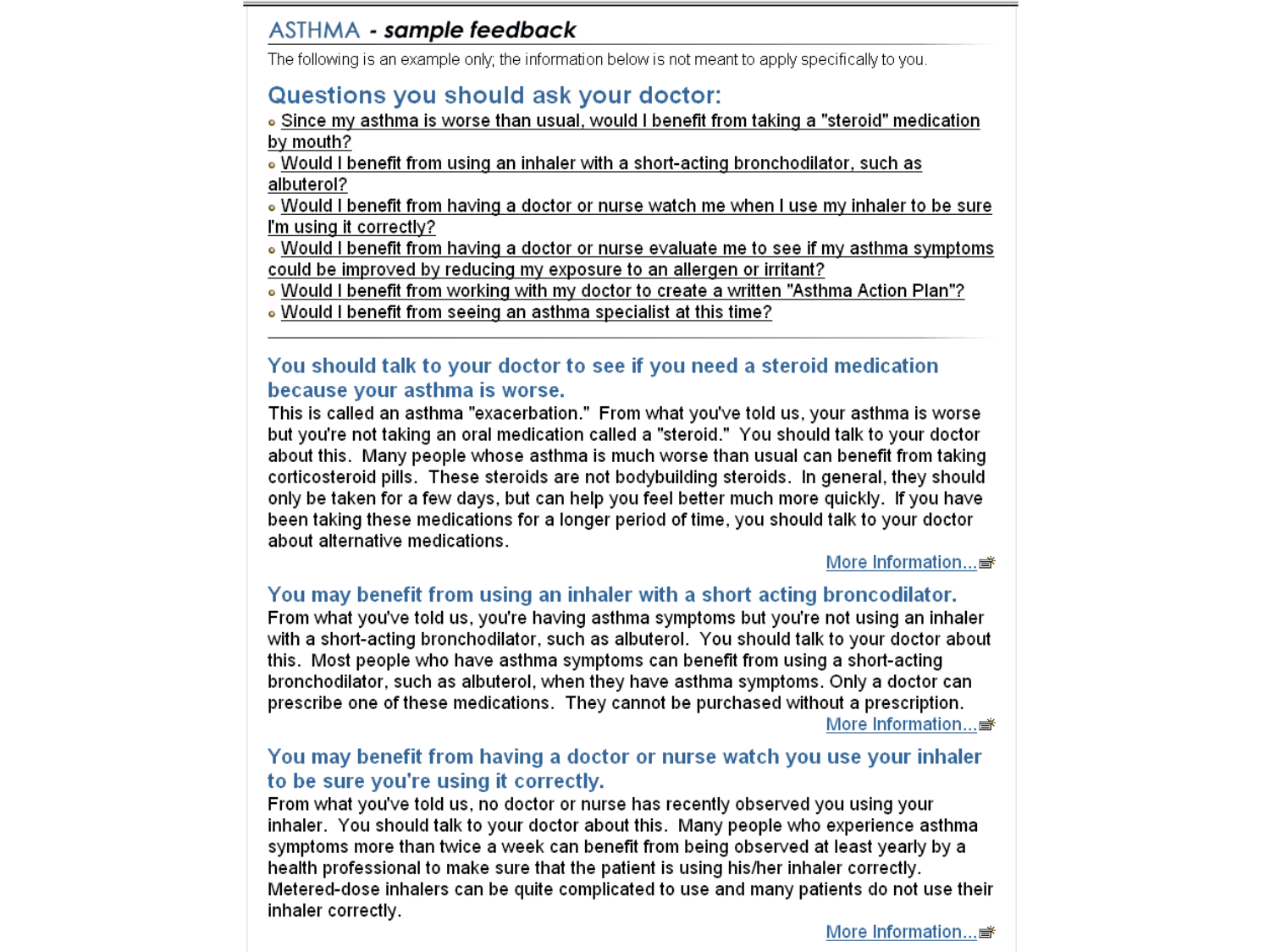

Supplement: Supplementary file 1 [file jmir_v9i1e3_app1.ppt]
